# Influence of AFP on surgical outcomes in non-B non-C patients with curative resection for hepatocellular carcinoma

**DOI:** 10.1007/s10238-022-00813-4

**Published:** 2022-03-15

**Authors:** Xiao-ping Tan, Kai Zhou, Qing-li Zeng, Yun-fei Yuan, Wei Chen

**Affiliations:** 1 Department of Emergency, The Second Affiliated Hospital of Guangzhou Medical University, Guangzhou, 510260 China; 2 Jiangxi Provincial People’s Hospital, Nanchang, 330006 Jiangxi China; 3 The 334 Hospital Affiliated to Nanchang University, Nanchang, 330024 Jiangxi China; 4 Department of Hepatobiliary Oncology, Sun Yat-sen University Cancer Center, Guangzhou, 510655 China; 5 Department of Colorectal Surgery, The Six Affiliated Hospital, Sun Yat-sen University, Guangzhou, 510655 China; 6 Guangdong Provincial Key Laboratory of Colorectal and Pelvic Floor Disease, The Sixth Affiliated Hospital of Sun Yat-sen University, Guangzhou, 510655 China; 7 Guangdong Research Institute of Gastroenterology, The Sixth Affiliated Hospital of Sun Yat-sen University, Guangzhou, 510655 China

**Keywords:** Hepatocellular carcinoma, Non-B non-C-HCC, AFP, Prognosis

## Abstract

To study the clinical and prognostic features of non-B non-C alpha-fetoprotein (AFP)(-)-hepatocellular carcinoma (HCC) (NBNC-AFP(-)-HCC) and the relationship between the prognostic features of HCC and hepatitis B virus surface antigen (HBsAg) status and AFP. We enrolled 227 patients who underwent hepatic resection for HCC between January 1998 and December 2007 at Sun Yat-sen University Cancer Center, all of whom were diagnosed with HCC by pathology. All patients were stratified into one of four groups (B-AFP(+)-HCC, B-AFP(-)-HCC, NBNC-AFP(+)-HCC, and NBNC-AFP(-)-HCC) according to AFP levels and HBsAg status. The clinicopathologic and survival characteristics of NBNC-AFP(-)-HCC patients were compared with those of all other three groups. Out of the 105 NBNC-HCC patients, 43 patients (40.9%) had AFP-negative HCC. There were some differences in factors between the B-AFP(+) and NBNC-AFP(-) patients, such as age, body mass index (BMI), diabetes, and ALT (*P* < 0.05). On univariate analysis, tumour size, secondary tumour, and portal invasion were prognostic factors for overall survival (OS) and disease-free survival (DFS) (*P* < 0.05). Cox multivariate regression analysis suggested that tumour size and tumour number (*P* < 0.05) were independent predictors. In addition, compared with the B-AFP(+)-HCC, B-AFP(-)-HCC, and NBNC-AFP(+)-HCC groups, the NBNC-AFP(-)-HCC patients had the best DFS (*P* < 0.05). Compared with the B-AFP(+)-HCC and NBNC-AFP(+)-HCC groups, the NBNC-AFP(-)-HCC patients had better OS (*P* < 0.05), and survival rates were similar to those of B-AFP(-)-HCC patients. NBNC-AFP(-)-HCC patients had a relatively favourable prognosis. It can serve as a useful marker in predicting the risk of tumour recurrence in the early stages.

## Background

Hepatocellular carcinoma (referred to as liver cancer or HCC) is the third most common cancer worldwide and the third leading cause of cancer-related death [1–4]. In China, the number and proportion of cases of non-B, non-C hepatocellular carcinoma (NBNC-HCC) have been increasing gradually [5]. The characteristics and prognosis of NBNC-HCC in these patients differ from those in patients with chronic hepatitis virus infection. Its related epidemiological factors include ageing, sex, alcoholism, diabetes, and metabolic syndrome [6, 7]. In addition, diabetes and obesity have been suggested as risk factors for NBNC-HCC in large cohort and case–control studies both with and without pre-existing non-alcoholic fatty liver (NAFLD), especially in terms of NBNC-HCC [8–11].

To date, the prognostic risk factors for NBNC-HCC have not been completely determined yet. Previous studies have shown that the prognosis of NBNC-HCC is associated with some factors, such as AFP level, liver fibrosis, tumour size, and ductal invasion [12, 13]. Therefore, investigating the clinical symptoms and prognosis of NBNC-HCC is helpful to understand the intrinsic mechanism underlying HCC progression. In this study, we selected 105 patients with NBNC-HCC and randomly selected 122 patients who were hepatitis B surface antigen (HBsAg)-positive and hepatitis C virus antibody (HCVAb)-negative (B-HCC). Our current study investigated the clinical characteristics and prognosis of NBNC-AFP(-)-HCC and explored the association among the expression of HBsAg, AFP, and survival prognosis.

## Patients and methods

### Materials

**Fig. 1:**
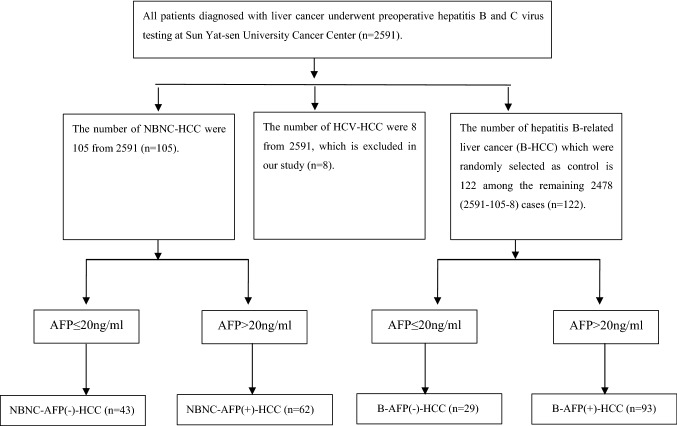
Consort diagram of the study selection process. A total of 227 patients were selected from 2591 HCC patients treated at Sun Yat-sen University Cancer Center between January 1998 and December 2007

This study selected cases from 2591 patients who underwent liver cancer resection at Sun Yat-sen University Cancer Center from January 1998 to December 2007. The inclusion criteria were that all cases should be patients who underwent liver tumour resection and postoperative pathological examination revealed HCC. All of patients were tested for HBV and HCV at admission. A total of 105 NBNC-HCC patients were screened, and 122 patients from the remaining 2486 non-NBNC-HCC patients were randomly selected as controls (Fig. 1). According to their expression of AFP and HBsAg, we divided the 227 patients into four groups (the negative standard was set as AFP ≤ 20 ng/ml), the NBNC-AFP(-)-HCC group (who were HBsAg-negative, HCVAb-negative, and AFP-negative, *n* = 43), NBNC-AFP(+)-HCC group (who were HBsAg-negative, HCVAb-negative, and AFP-positive, *n* = 62), B-AFP(+)-HCC group (who were HBsAg-positive, HCVAb-negative, and AFP-positive, *n* = 93), and B-AFP(-)-HCC group (who were HBsAg-positive, HCVAb-negative, and AFP-negative, *n* = 29). Postoperative treatment of HCC includes transcatheter arterial chemoembolization (TACE), secondary surgery, radiofrequency ablation (RFA), portal vein chemotherapy (PVC), and radiation therapy.

### Definition of clinical pathological characteristics

The patients’ vital signs or assay indexes were recorded according to their test results after their admission to the hospital and before surgery. Influencing factors included age, body mass index (BMI), metabolic syndrome (pathoglycaemia, abnormal blood pressure, and dyslipidaemia), ALT, AST, S/T, size of tumour, secondary tumour, incisal edge, portal invasion, and pathological grade.

In this study, the diagnosis of metabolic syndrome was based on the 2005 International Diabetes Federation Standard, and patients who met three of the following five conditions were diagnosed with metabolic syndrome: (1) obesity: waistline > 90 cm (men) or > 80 cm (women) and/or BMI > 25 kg/m^2^; (2) serum triglyceride (TG) ≥ 1.7 mmol/L or previously diagnosed with hypertriglyceridemia; (3) high-density lipoprotein cholesterol (HDL-C) < 1.03 mmol/L (men) or < 1.29 mmol/L (women); (4) arterial blood pressure ≥ 130/85 mmHg or previously diagnosed with hypertension; and (5) fasting plasma glucose (FPG) ≥ 5.6 mmol/L or previously diagnosed with type II diabetes.

### Follow-up and statistical analysis

Patients were followed up until January 2012. The interval of tumour recurrence was calculated in months, while survival time was calculated in years. The cut-off point for disease-free survival (DFS) time was tumour recurrence or metastasis confirmed on initial postoperative imaging, such as computed tomography (CT), B-ultrasound, or magnetic resonance imaging (MRI). Overall survival (OS) was calculated as the interval between the date of surgery and the date of death or the date of the last follow-up. Survival rates were calculated using Kaplan–Meier analysis, and the log-rank test was used for univariate analysis. The variables that were found to be significant in the univariate analysis were introduced into the Cox model for further multivariate analysis. *P* < 0.05 was considered statistically significant. We used SPSS13.0 for the statistical analysis.

## Results

Clinical pathological characteristics of NBNC-AFP(-)-HCC patients.

**Fig. 2:**
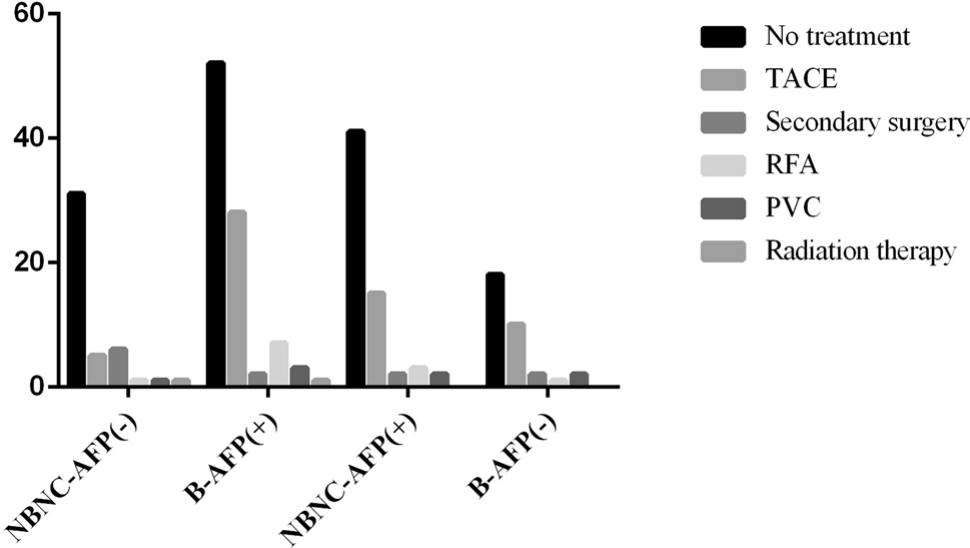
Comparison of the number of patients who underwent postoperative treatment in different groups

As shown in Fig. 1, there were 43 NBNC-AFP(-)-HCC patients and 62 NBNC-AFP(+)-HCC patients, which accounted for 1.6% (43/2591) and 2.4% (62/2591) of all patients, respectively. The Chi-squared test was used to compare differences in clinical pathological characteristics between the NBNC-AFP(-)-HCC group and the other three groups (Table 1). Consequently, there were statistically significant differences in age, BMI, diabetes, metabolic syndrome, ALT, AST, tumour size, secondary tumour, portal invasion, postoperative treatment and pathological grade (*P* < 0.05) between the first groups (B-AFP(+) vs. NBNC-AFP(-)). Age, ALT, portal invasion, and pathological grade showed statistically significant differences in the third group (B-AFP(-) vs. NBNC-AFP(-)) (*P* < 0.05). There were significant statistical differences in AST, S/T, tumour size, secondary tumour, portal invasion, postoperative treatment and pathological grade between NBNC-AFP(-)-HCC patients and NBNC-AFP(+)-HCC patients. In addition, the number of patients who underwent the postoperative treatment in the different groups is shown in Fig. 2.

**Table 1:** Comparison of clinicopathological features of NBNC-AFP(-)-HCC patients

Characteristic	NBNC-AFP(-)	B-AFP(+)	*P*	NBNC-AFP (+)	*P*	B-AFP(-)	*P*
Age (years)			0.004		0.155		0.024
≥ 50	32	45		53		14	
< 50	11	48		9		15	
Drink			0.111		0.695		0.440
Yes	9	10		15		4	
No	34	83		47		25	
BMI			0.015		0.156		0.612
> 25 kg/m^2^	11	9		9		9	
≤ 25 kg/m^2^	32	84		53		20	
Diabetes			0.003		0.097		0.109
Yes	11	7		8		3	
No	32	86		54		26	
Metabolic syndrome			0.019		0.684		0.650
Yes	6	3		7		3	
No	37	90		55		26	
ALT			< 0.001		0.305		0.002
> 40 U/L	12	62		12		19	
≤ 40 U/L	31	31		50		10	
AST			0.001		< 0.001		0.104
> 40 U/L	14	60		59		15	
≤ 40 U/L	29	33		3		14	
S/T			0.088		< 0.001		0.396
> 1	28	46		58		16	
≤ 1	15	47		4		13	
Tumour size (cm)			0.011		0.006		0.664
< 5	20	23		13		15	
≥ 5	23	70		49		14	
Secondary tumour			0.026		0.015		0.506
Yes	5	27		20		2	
No	38	66		42		27	
Portal violation			0.025		< 0.001		< 0.001
Yes	0	10		15		8	
No	43	83		47		21	
Pathological grade			0.007		0.001		0.022
I	7	3		0		0	
II–IV	36	90		62		29	
Postoperative treatment			< 0.001		0.009		0.370
No	31	29		29		18	
Yes	12	64		33		11	

There were statistically significant differences in age, BMI, diabetes, metabolic syndrome, ALT, AST, tumour size, secondary tumour, portal violation, postoperative treatment and pathological grade between NBNC-AFP(-) and B-AFP(+). In addition, the portal invasion and pathological grade were found to be statistically significant differences in other two groups

### DFS of NBNC-AFP(-)-HCC patients

Univariate analysis of all 227 HCC patients in this study showed that factors influencing postoperative DFS included AST, S/T, size of tumour, secondary tumour, and portal invasion (*P* < 0.05) (Table 2). After introducing these statistically significant indexes in the univariate analysis to the Cox model for multivariate analysis, it was shown that the size of primary tumour, secondary tumour, and portal invasion were all independent influencing factors for postoperative DFS (*P* = 0.008, *P* < 0.001, and *P* = 0.006, respectively) (Table 3). Univariate analysis of 43 NBNC-AFP(-)-HCC patients revealed that the secondary tumour was a factor that could influence postoperative DFS (*P* = 0.016) (Table 4).

**Table 2:** Univariate analysis of DFS and OS after HCC resection

Characteristic	DFS (*P*)	OS (*P*)
Age	0.294	0.863
BMI	0.897	0.917
Drink	0.408	0.478
Diabetes	0.925	0.174
Metabolic syndrome	0.156	0.923
ALT	0.708	0.204
AST	0.049	0.216
S/T	0.003	0.216
Tumour size	< 0.001	< 0.001
Secondary tumour	< 0.001	< 0.001
Portal violation	0.011	< 0.001
Pathological grade	0.230	0.193

A total of twelve clinical variables were screened as prognostic factors to predict OS and DFS using a univariate analysis

**Table 3:** Multivariate analysis of disease-free survival rate and overall survival rate after HCC resection

Characteristic	DFS		OS	
	95.0% CI for Exp(B)	*P*	95.0% CI for Exp(B)	*P*
AST	0.761–1.820	0.464	0.902–2.142	0.136
S/T	–	–	0.934–2.237	0.099
Tumour size	0.294–0.831	0.008	0.365–0.981	0.042
Secondary tumour	1.712–4.162	< 0.001	1.076–2.398	0.020
Portal violation	1.260–3.821	0.006	0.775–2.020	0.359

A total of five clinical variables were screened as prognostic factors to predict OS and DFS using a multivariate analysis

**Table 4:** Univariate analysis of postoperative DFS and OS in patients with NBNC-AFP(-)-HCC

Characteristic	DFS (*P*)	OS (*P*)
Age	0.057	0.985
BMI	0.822	0.419
Drink	0.408	0.397
Diabetes	0.118	0.711
Metabolic syndrome	0.467	0.635
ALT	0.668	0.649
AST	0.695	0.292
S/T	0.848	0.249
Tumour size	0.162	0.528
Secondary tumour	0.016	0.959
Portal violation	0.500	0.510
pathological grade	0.165	0.365

A total of twelve clinical variables were screened as prognostic factors to predict OS and DFS using a univariate analysis in NBNC-AFP(-)-HCC group

**Fig. 3:**
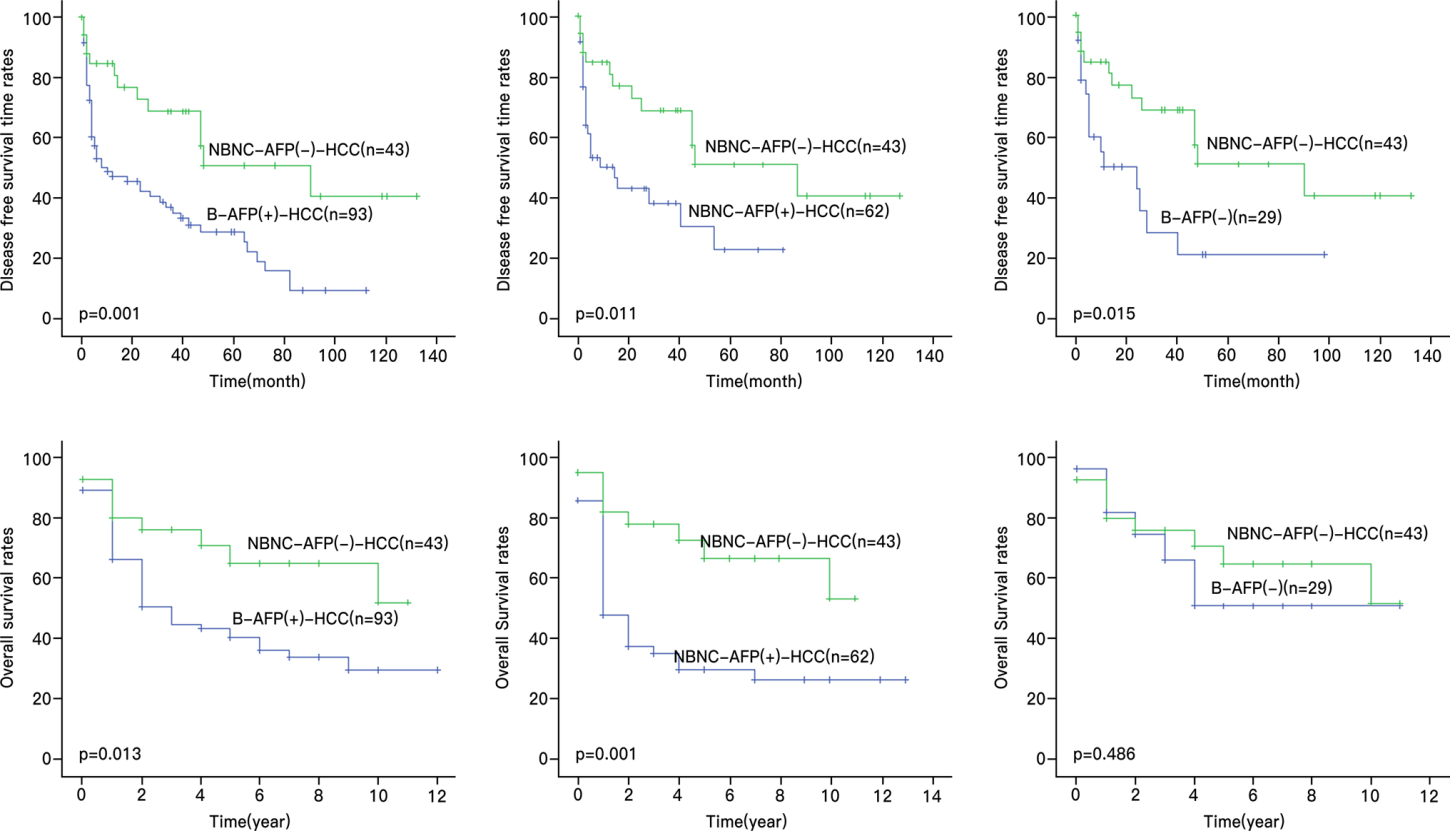
Comparison of clinical outcomes between the NBNC-AFP(-)-HCC patients and other groups

The Kaplan–Meier analysis showed that NBNC-AFP(-)-HCC patients had longer DFS than the other three groups (Fig. 3). With the follow-up time ranging from 1 to 132 months, the median DFS of the four groups (NBNC-AFP(+)/(-)-HCC groups and B-AFP(+)/(-)-HCC groups) was 15 months, 90 months, 10 months, and 24 months, respectively, and their corresponding 1-year DFS was 50.3%, 84.6%, 47.3%, and 50.1%, while their 2-year DFS was 43.1%, 72.9%, 42.3%, and 42.9%. The 3-year DFS rates were 38.3%, 68.8%, 35.3%, and 21.5%, respectively; the 5-year DFS rates were 23%, 51%, 29%, and 21.5%; and the 10-year DFS rates were 0, 40.8%, 9.7%, and 0. Among these three groups of case controls, the NBNC-AFP(-)-HCC group was found to be significantly different from the B-AFP(+)-HCC, NBNC-AFP(+)-HCC, and B-AFP(-)-HCC groups in terms of patients’ DFS duration (*P* = 0.001, *P* = 0.011, and *P* = 0.015, respectively). The above data show that the 1-year, 2-year, 3-year, 5-year, and 10-year DFS rates of NBNC-AFP(-)-HCC patients were 84.6%, 72.9%, 68.8%, 51%, and 40.8%, respectively, and the patients’ median DFS duration was 90 months. After 10 years, 40.8% of NBNC-AFP(-)-HCC patients had no tumour recurrence, which outperformed the other three groups in terms of DFS (Table 5).

**Table 5:** Comparison between groups in terms of the DFS and OS

	1	3	5	10
*DFS(year)*				
NBNC-AFP(-)	84.6	68.8	51	40.8
B-AFP(+)	47.3	35.3	29	9.7
NBNC-AFP(-)	84.6	68.8	51	40.8
NBNC-AFP(+)	50.3	38.3	23	0
NBNC-AFP(-)	84.6	68.8	51	40.8
B-AFP(-)	50.1	21.5	21.5	0
*OS(year)*				
NBNC-AFP(-)	80.1	76.1	65.1	52.1
B-AFP(+)	66.4	44.6	40.5	29.7
NBNC-AFP(-)	80.1	76.1	65.1	52.1
NBNC-AFP(+)	83.9	34.2	28.9	25.7
NBNC-AFP(-)	80.1	76.1	65.1	52.1
B-AFP(-)	82.2	66.5	51.5	51.5

NBNC-AFP(-)-HCC patients had significantly better DFS and OS than NBNC-AFP(+)-HCC and B-AFP(+)-HCC patients

### OS of NBNC-AFP(-)-HCC patients

The univariate analysis showed that some factors were associated with OS (*P* < 0.05), such as the size of the primary tumour, secondary tumour, and portal vein invasion (Table 2), while Cox multivariate analysis indicated that the size of the primary tumour (*P* = 0.042) and secondary tumour (*P* = 0.020) were both independent influencing factors for postoperative OS (Table 3). In this study, no relevant factors affecting postoperative OS in NBNC-AFP(-)-HCC patients were found (Table 4).

Kaplan–Meier analysis revealed that NBNC-AFP(-)-HCC patients had significantly better OS than NBNC-AFP(+)-HCC and B-AFP(+)-HCC patients, but there was no significant difference compared with that in B-AFP(-)-HCC patients. The median survival times of patients in these four groups (NBNC-AFP(+)/(-)-HCC groups and B-AFP(+)/(-)-HCC groups) were 1 year, 10 years, 2 years, and 4 years, and their 1-year OS rates were 83.9%, 80.1%, 66.4%, and 82.2%, respectively. The 3-year OS was 34.2%, 76.1%, 44.6%, and 66.5%; the 5-year OS was 28.9%, 65.1%, 40.5%, and 51.5%; and the 10-year OS was 25.7%, 52.1%, 29.7%, and 51.5% (follow-up ranged from 1 to 11 years). The median survival time of NBNC-AFP(-)-HCC patients was 10 years, and 52.1% of postoperative patients survived for at least 10 years (Table 5).

## Discussion

The digestive system cancers are the most common malignant tumour and the leading cause of cancer-related deaths worldwide [14, 15]. The HBV and HCV are the main causes of HCC in China and Japan, respectively [16–18]. With the widespread use of the vaccine, the number of children and carriers of HBV have significantly declined in recent years [19]. Approximately, 5%–20% of HCC patients who are negative for both HBsAg and HCVAb, so-called “NBNC-HCC”, have recently tended to increase [20, 21].

To date, the underlying causes of NBNC-HCC are not yet completely understood. Our research identified that the aetiology of NBNC-AFP(-)-HCC in patients in China was related to obesity, diabetes, metabolic syndrome, and NAFLD, which is consistent with existing reports [22]. Some factors are associated with non-alcoholic steatohepatitis (NASH) and liver fibrosis, such as age, obesity, hypertension, type II diabetes, ALT, S/T > 1, and decreased platelet count [23, 24]. A series of studies confirmed that obesity and diabetes were independent risk factors for HCC [25]. The aetiological basis of such findings was insulin resistance (IR), which was the shared pathogenic factor for obesity, diabetes, hypertension, and dyslipidaemia for causing fatty liver, as well as a factor during the progression of liver disease from steatohepatitis, liver fibrosis, and cirrhosis to HCC [26]. In addition, this study did not exclude alcoholic patients. There was no significant difference between the three groups of patients who were included according to the standard of excessive drinking [drinking alcohol > 280 g/week (men) or > 140 g/week (women)], which indicated that there may be no strong correlation between NBNC-AFP(-)-HCC and alcohol consumption.

Meanwhile, no significant difference existed in age, BMI, history of drinking, metabolic syndrome, or diabetes between NBNC-AFP(-)-HCC and NBNC-AFP(+)-HCC, indicating that these two types shared a similar aetiology. However, patients with NBNC-AFP(-)-HCC had special clinical characteristics, which can be seen in the existence of significant differences in size of tumour, pathological type, secondary tumour, and portal invasion. The underlying pathological mechanism might be that the lack of expression of AFP in the absence of hepatitis B and C virus infections could lead to good clinical pathological characteristics. Shian et al. reported that HCC patients who expressed high levels of AFP (> 200 ng/ml) had larger tumour diameters (> 5 cm), increased pathological grading (II–IV), and increased portal invasion (IIIA–IV HCC) than those who expressed low levels of AFP (< 200 ng/ml). The high expression of AFP would also leads to an increase in the recurrence rate in HCC patients [27]. This was consistent with our findings and further confirmed that AFP was an important factor affecting survival in HCC [28–30]. Meanwhile, multivariate analysis showed that the size of primary tumour, secondary tumour, and portal invasion were all independent influencing factors for DFS, while the size of tumour and secondary tumour were independent influencing factors for OS. Hence, we believe that the special clinical characteristics of NBNC-AFP(-)-HCC patients directly lead to their good survival prognosis.

The univariate analysis showed that the secondary tumour was the factor that influenced DFS, while no factors were found to affect OS. Our research suggests that being AFP- and HBsAg-negative at the same time could indicate a low recurrence rate and that there was a partial difference in terms of the OS. In addition, NBNC-AFP(-)-HCC patients had lower recurrence rates and higher OS than NBNC-AFP(+)-HCC patients*.* The pathological mechanism of the good prognosis for NBNC-AFP(-)-HCC might be the negative expression of AFP in the absence of hepatitis B and C virus infections. In China, most HCC progresses on the basis of chronic hepatic damage, and hepatitis virus can not only induce the occurrence of tumours, but also promote the recurrence and metastasis of HCC [31, 32]. Meanwhile, the high expression of AFP also plays an important role in the progression of HCC and could serve as a useful symbol for predicting the risk of early HCC recurrence postoperatively.

Some factors have been found to be associated with the prognosis of NBNC-HCC, such as MicroRNA-96-5p, PNPLA3 and TM6SF2 polymorphisms [33, 34]. AFP values were generally lower in NBNC-HCC patients than in B-HCC patients due to their unaffectedness by hepatitis virus. According to some previous studies, HBX can directly upregulate the expression of AFP by binding to and activating the promoter of the AFP gene [35, 36]. In addition, the better prognosis of NBNC-HCC patients than HBV-HCC patients may be related to age and CD34 [37, 38]. Current studies have shown that CD34, a marker factor of vascular endothelial cells, is closely related to the angiogenesis of in HCC. CD34 has a synergistic effect with vascular endothelial growth factor (VEGF), which together promote the angiogenesis and cell proliferation of HCC, and then lead to the metastasis of liver cancer and the formation of tumour thrombi, thus affecting the prognosis.

## Conclusions

In summary, NBNC-AFP(-)-HCC was correlated with ageing, obesity, diabetes, and metabolic syndrome, suggesting that the aetiology of NBNC-AFP(-)-HCC was related to NAFLD. Patients with NBNC-AFP(-)-HCC had the lowest recurrence rate, which was associated with their special clinical characteristics and serological features. However, there are some limitations of our study, such as a retrospective single-centre study and a lack of analysis of hepatitis C-related liver cancer patients in the control group.

## Data Availability

The data used and/or analysed during the current study are available from the corresponding author on reasonable request.

## Abbreviations

AFP: Alpha-fetoprotein
ALD: Alcoholic hepatitis
ALT: Alanine aminotransferase
AST: Aspartate aminotransferase
B-HCC: Hepatitis B virus related hepatocellular carcinoma
DFS: Disease-free survival
FPG: Fasting plasma glucose
HBV: Hepatitis B virus
HCC: Hepatocellular carcinoma
HCV: Hepatitis C virus
HDL-C: High-density lipoprotein cholesterol
IR: Insulin resistance
NAFLD: Non-alcoholic fatty liver
NASH: Non-alcoholic steatohepatitis
NBNC-HCC: Non-B non-C HCC
OS: Overall survival
S/T: Aspartate aminotransferase/Alanine aminotransferase
TG: Triglyceride
